# Adverse Effects of Toxic Metal Pollution in Rivers on the Physiological Health of Fish

**DOI:** 10.3390/toxics10090528

**Published:** 2022-09-08

**Authors:** Huong Thi Thuy Ngo, Thanh Dinh Nguyen, Tien Thi Hanh Nguyen, Thao Thanh Le, Dinh Quoc Nguyen

**Affiliations:** 1 Faculty of Biotechnology, Chemistry and Environmental Engineering, Phenikaa University, Hanoi 12116, Vietnam; 2 Bioresource Center, Phenikaa University, Hanoi 12116, Vietnam; 3 Economic Geology and Geomatics Department, Vietnam Institute of Geosciences and Mineral Resources, Hanoi 12109, Vietnam

**Keywords:** metal accumulation, biomarkers, fish, toxicity, health, Nhue–Day River basin

## Abstract

Toxic metal pollution influences the lives of diverse aquatic organisms and humans who consume contaminated aquatic products. However, its potential impacts on aquatic organism health and, thus, ecological health, have been neglected in many regions. This research was carried out to contribute to filling that knowledge gap. Three freshwater fish species in the Nhue–Day River basin, Vietnam, have been chosen to study the bioaccumulation of metals (Zn, Cu, Pb, and Cd) in the tissues (livers, kidneys, gills) and their effects on fish physiological health (changes in the oxidative-GST activity, and physiological biomarkers-energy reserves, respectively) from 2013 to 2017. The extensive results revealed significant spatial and temporal variations in metal concentrations in tissues of common carp (*Cyprinus carpio*), silver carp (*Hypothalmic molitrix*), and tilapia (*Oreochromis niloticus*), and well correlated to their concentration in the water (*p* < 0.05). Fish bioaccumulated metals in the following order: Zn > Cu > Pb > Cd, with more in the kidneys and livers (spring and summer) than in other tissues. Metal accumulation in *O. niloticus* and *C. carpio* was higher than in *H. molitrix*. Biomarker responses (except for glycogen variation) were also higher during warm seasons. Changes in metal levels in water and fish tissues caused variations in biomarkers in the respective fish tissues, particularly in the livers, as demonstrated by significant correlations of metal concentrations in water and fish tissues to biochemical and physiological responses (*p* < 0.05). The findings suggest that metal pollution in the river basin adversely impacts the physiological health of both wild and cultured fish. Seasonal shifts in the levels of metal accumulation and biomarkers could be connected to species-specific differences in physiology and the levels of metals in environments. This biomarker set is simple but effective in assessing the impact of metal pollution on fish health and, hence, the aquatic ecosystem. This is one of the first biomonitoring studies to assist in designing better water management strategies for the Nhue–Day River basin.

## 1. Introduction

In recent decades, trace metals from mining, industrial, agricultural, and domestic wastes have polluted aquatic environments. Due to their high toxicity, non-degradability, and propensity to accumulate in the food chain, trace metals can pose a significant threat to aquatic organisms [1]. Once accumulated in the body, metals may induce oxidative stress by producing reactive oxygen species (ROS), including superoxide radical (O_2_^•−^), hydrogen peroxide (H_2_O_2_), hydroxyl radicals (OH^•^), and singlet oxygen (^1^O_2_) [2], consequently, affecting physiological health [3]. ROS can oxidize proteins, lipids, and nucleic acids unless detoxified, causing cell damage or death [4]. Increased oxidative stress by trace metals affects fish growth, development, and reproduction by alternating metabolic, physiological, and biochemical processes [5]. It can also cause neurodegeneration, thyroid dysfunction, fish larval malformations, inflammation, development, and reproductive disorders [6,7,8]. Biomarkers in wild fish are essential in assessing chemical pollution’s impact on aquatic ecosystems. This will help bridge the gap and establish links between ecological and chemical assessments regarding causality [9]. 

Organisms have developed enzymatic and non-enzymatic antioxidant defense systems to combat metal-caused ROS. Antioxidants convert ROS to harmless metabolites, protecting cellular metabolism and functions [10]. There have been several key antioxidant enzymes, such as catalase (CAT) or glutathione peroxidase (GPx), which act on H_2_O_2_; glutathione-S-transferases (GSTs), which metabolize lipid hydroperoxides in response to ROS production, in addition to xenobiotic biotransformation; and glutathione reductase, which is associated with the maintenance of reduced glutathione (GSH) [11]. These molecules or ligands are often used as biomarkers in monitoring aquatic ecosystems because changes in cellular antioxidant defenses reflect exposure to contaminants and their toxicity [12].

Growth, reproduction, and basal metabolism consume most of an organism’s energy. However, under environmental stresses caused by toxicants, such as metals, organisms must increase their energy expenses for detoxification and compensate for the metabolic cost of defense [13]. During metal stress, animals mobilize energy reserves, such as carbohydrates, lipids, and proteins, to meet these increased energy demands [3,14]. Therefore, changes in energy reserves can be used as a non-enzymatic biomarker for metal stress [3,13]. Some species, such as freshwater mussels (*Anodonta anatina*) exposed to Cd at environmental-like levels [3] and freshwater snails (*Galba truncatula*) exposed to municipal wastewater [15] had decreased glycogen storage. Reduced protein, glycogen, and lipid contents were also observed in terrestrial isopods and beetles [14].

At the top of the aquatic food chain, fish can accumulate metals and transmit them to humans through consumption, causing chronic and acute diseases [16]. Therefore, fish are often used to monitor pollution in aquatic systems [17]. The Nhue–Day River basin, located southwest of the Red River Delta, provides water for agriculture, aquaculture, and water supply for millions of people in five northern provinces of Vietnam. However, the basin’s environment has been degraded by urbanization and socioeconomic development. Many industrial zones, traditional craft villages, and factories near the two rivers negatively impact the rivers’ water quality [18]. According to Nguyen et al. [19], the Nhue river was moderately polluted by As (0.2–131 μg/L) and Hg (0.11–4.1 μg/L), and slightly polluted by Cd (2.1–18 μg/L). Increased pollution of these rivers has drawn public attention in recent years, and the system has been categorized as one of the most severely polluted river systems in Vietnam [20].

Until now, the limited information on Nhue–Day River metal pollution was mostly based on chemical analyses of water, sediment, and fish [21,22,23,24,25]. The scarcity of research on the potential effects of metals on aquatic animals’ health requires urgent metal biomonitoring of the system. Biomonitoring studies can provide a more sensitive assessment of pollution status by incorporating bioavailability and intrinsic toxicity, as well as detecting substances at low levels [26]. Therefore, this study aimed to investigate (1) variations of trace metal levels (i.e., Zn, Cu, Cd, and Pb) in the basin and the tissues (livers, kidneys, and gills) of common carp (*Cyprinus carpio* L), silver carp (*Hypophthalmic molitrix*), and tilapia (*Oreochromis niloticus);* and (2) the impacts of these metals on oxidative and physiological biomarkers’ responses (GSTs, total protein, and glycogen levels) of aquatic organisms along the Nhue–Day River basin. These fish species (from rivers and aquaculture ponds using rivers’ water) were chosen due to their significant economic interest, commonly raised in the river basin, and essential food sources for people in northern Vietnam [21]. Furthermore, the relationships between metal concentrations in water and their bioaccumulation in fish tissues were investigated, as were the relationships between metal levels and GST activities, glycogen, and protein contents in respective tissues.

## 2. Materials and Methods

### 2.1. Study Area, Selected Organisms, and Sampling

**Figure 1:**
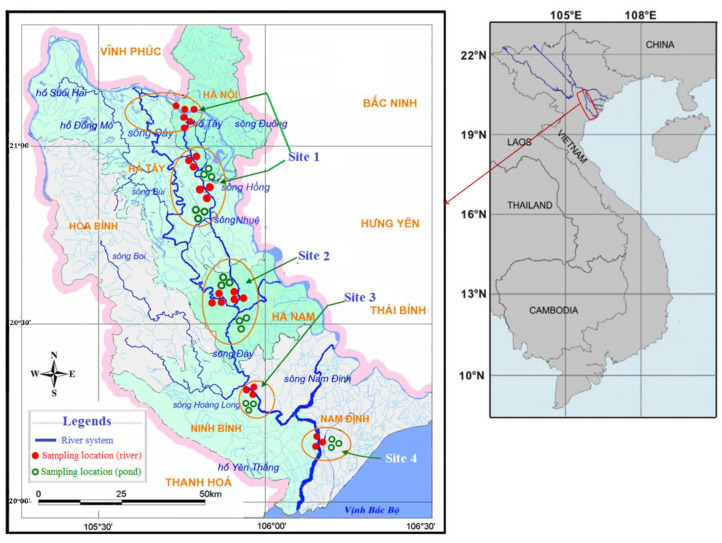
The study area map depicts the Nhue–Day River basin as well as sampling locations. Closed red dots represent river sampling locations, while open green dots represent aquaculture pond locations.

The study area is located along the Nhue River, from Hanoi Capital (Site 1, at the confluence with the Red River) to Ha Nam Province (Site 2, at the confluence with the Day River) and downstream of the Day River in Ninh Binh Province (Site 3, at the confluence with the Hoang Long River) and Nam Dinh Province (Site 4, after the confluence with the Nam Dinh River). This region is in the northern plains at 20°–21°20′ north latitude and 105^o^–106^o^30′ east longitude (Figure 1).

To achieve the stated research objectives, an average of 38 water and 30 fish samples from each season were collected from the rivers (24 locations) and aquaculture ponds (18 locations) at four study sites during four seasons between 2013 and 2014: spring (April), summer (July), autumn (September), and winter (December). Between 2015 and 2017, additional information regarding the status of aquaculture and fish consumption in the river basin was gathered. Fish were collected directly from the rivers or aquaculture ponds that used water from the Nhue–Day River system. The collected species were returned to the laboratory alive in rich-oxygen containers and anesthetized before being dissected for gills, livers, and kidneys. About 10–20 mg wet weight (wwt.) (gills) or 5–10 mg wwt. (livers and kidneys) were stored at −80 °C in an Eppendorf tube containing 300 μL of Dulbecco’s phosphate buffered saline (DPBS) for later analysis of GST activities, glycogen, and protein contents. Another 20–100 mg wwt. of each dissected tissue was also taken for trace metal analysis.

All the chemicals used in the analysis were of the analytical grade (Merck, Darmstadt, Germany). Analytical containers and tools were washed with tap water before being soaked in 1:1 conc. HNO_3_ for 24 h (h), rinsed with double-distilled water and dried before use.

### 2.2. Metal Analysis

Water and tissue samples, including a blank (only reagents) and a reference material sample, were digested in 2.5 mL of aqua regia (conc HNO_3_ and conc HCl, *v/v* 1:3) at room temperature for 24 h. Then, 200 μL of H_2_O_2_ was added to each test tube and left at room temperature for another 5 h before being digested in a digestion box (bio-carrier) at 120 °C for at least 5 h until completely digested. After that, the digested samples were diluted with double distilled water up to 20 mL and filtered through a cellulose syringe filter with a pore size of 45 μm. Samples were then ready for metal analysis by inductively coupled plasma mass spectrometry (ICP–MS; Varian Ultra Mass 700, Victoria, Australia). The detection limits for Cu and Zn were 1 μg L^−1^, and for Cd and Pb were 0.001 μg L^−1^. The analytical method was validated with the certified standard reference material DOLT-4 (Dogfish liver). Procedural blanks of aqua regia were included in every batch of samples and were below the detection limits. The results were reported in mg L^−1^ for water samples and mg kg^−1^ wet weight for fish, except for Cd (µg kg^−1^ wwt).

### 2.3. Biomarker Measurements

#### 2.3.1. Glutathione S-Transferases (GSTs) Activity Assay

GST activity was determined according to Habig et al. [27] using 1-chloro 2,4-dinitrobenzene as a substrate with some modifications. The samples were thawed on ice, homogenized, and centrifuged at 9205 rpm for 15 min at 4 °C (5417R Eppendorf centrifuge, Germany). After removing the supernatant, the pellets were resuspended in 200 μL of DPBS buffer and centrifuged again to collect as much enzyme as possible. The combined supernatant (from two centrifugation times) was used for the GSTs and protein assay. The substrate (reaction solution) was a mixture of 100 mM DPBS buffer (pH 6.5), 200 mM GSH and 100 mM CDNB. The reaction was started by mixing 0.95–0.98 mL of the reaction mixture with 0.02–0.05 mL of samples, and the absorbance was measured every minute (for 8 min) at 340 nm using a Thermo Scientific^TM^ Biomate spectrophotometer. A blank sample containing 1 mL of the substrate was included. The specific activities of GSTs were calculated and expressed as nmoles of GSH-CDNB conjugate formed/min/mg protein.

#### 2.3.2. Glycogen Quantification

Glycogen levels in the tissues were determined by a modified phenol-sulfuric acid method [28,29]. The glycogen stock solution was used to make a series of standard concentrations. The samples were digested by adding 300 μL of KOH 30% to each tissue sample and standard, then shaking for 20 min in a water bath at 100 °C. The digested samples were cooled on ice before mixing with 200 μL ethanol 95% and shaken in a water bath at 100 °C for another 15 min. Glycogen levels in each tissue and standard samples were determined at 595 nm using 96-well plates, which contained 25 μL of samples, 25 μL of phenol 8%, and 140 μL of concentrated H_2_SO_4_ in each well.

#### 2.3.3. Protein Quantification

The total protein contents in some tissues were determined using the Bradford method [30]. In brief, aliquots (50 µL) of the combined supernatants or standard (bovine serum albumin-BSA) were diluted with 950 µL of NaOH (0.01 N) solution and vortexed for 10 s before incubating at 60 °C for 30 min. Then, 20 µL of each sample mixture was dispensed into a 96-well microtiter plate (3 replicates); 150 mL of 3x diluted Bradford solution was quickly added to each well. The same procedure was applied to make the standard curve. After 5 min up to 1 h, the optical density signals were measured at 595 nm, with a reference filter of 495 nm, using a microplate reader (Model 680, Bio-Rad Laboratories, Inc., Hercules, CA, USA). The protein content was expressed as μg g^−1^ wet weight.

### 2.4. Data Analysis

All data are presented as mean values ± standard error of the mean (SEM). Before proceeding with any statistical analysis, the Shapiro–Wilk test was used to ensure that all data followed a normal distribution. Differences in metal concentration in water, metal bioaccumulation, and biomarkers in fish across four sites and four sampling seasons were tested by the two-way analysis of variance (ANOVA), followed by the Tukey–Kramer multiple comparisons test. If the data set did not meet the normality assumptions, Bonferroni tests were used to identify significant differences. The Pearson and Spearman correlation tests were used to detect the relationship between metal levels in water and fish tissue and between tissue biomarkers and metal levels. Statistical significance was assigned at *p* < 0.05, *p* < 0.01, and *p* < 0.001. All statistical analyses were performed by GraphPad Prism version 9.1.1 for macOS and R Studio.

## 3. Results

### 3.1. Spatial and Temporal Variation of Metal Pollution in Water

**Figure 2:**
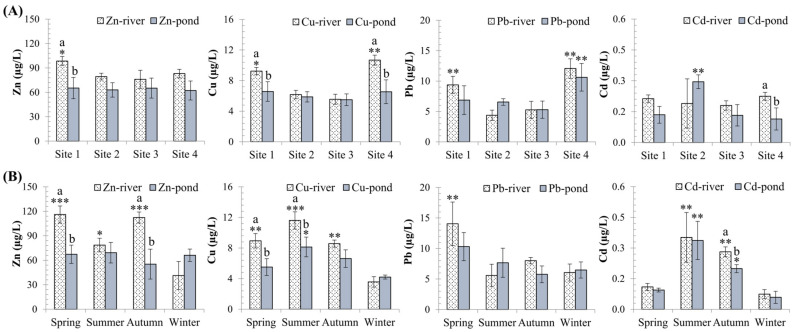
Spatial (**A**) and seasonal variations (**B**) of metals in water (µg/L). Significant differences between sampling sites and times in comparison to the lowest value (*) are indicated (mean ± SEM, *n* = 10–20, * *p* < 0.05, ** *p* < 0.01, *** *p* < 0.001). Different letters (a > b) indicate that values from the ponds and the rivers at the same locations (**A**) or at the same times (**B**) are significantly different (*p* < 0.05; ANOVA followed by Tukey–Kramer multiple comparisons test).

In terms of spatial variation, there was no discernible difference in Cd levels in the river, with a higher tendency at sites 1 and 4, whereas site 2 had the highest Cd concentrations in the aquaculture ponds (*p* < 0.001; Figure 2A). Cu and Pb levels in rivers were statistically higher at sites 1 and 4 than at sites 2 and 3 (*p* < 0.05; Figure 2A). Zn levels in river water were highest at site 1 (*p* < 0.05; Figure 2A).

Summer had the highest Cd concentrations in the water, followed by autumn, while winter and spring had the lowest concentrations (*p* < 0.0001; Figure 2B). Zn levels were lowest in the winter (*p* < 0.0001), but no differences were found in pond water (*p* > 0.05). The highest Pb concentrations were observed in the spring (*p* = 0.04), with no difference found in other seasons (Figure 2B).

### 3.2. Metal Accumulation in Fish Tissues

The average metal concentration in all three fish tissues followed the decreasing order: Zn > Cu > Pb > Cd (Table 1). Metals tended to accumulate more in the kidneys than in the livers and gills for Zn, Pb, and Cd, but to varying degrees: kidneys > livers ≥ gills for Zn; kidneys ≥ livers ≥ gills for Pb; and kidneys >> livers > gills for Cd. On the other hand, Cu accumulated much more in the fish’s liver than in their kidney or gills (*p* < 0.05). Surprisingly, the concentrations of Zn in all tissues of *C. carpio* were significantly higher than those of the other two species (*p* < 0.01; Table 1). However, *O. niloticus* accumulated significantly more Cu in its livers than *C. carpio* and *H. molitrix* (*p* < 0.01). Pb levels in the tissues of all tested fish species did not differ significantly (*p* > 0.05). Cd levels were higher in the kidneys than in other tissues, particularly the gills (*p* < 0.05). No annual differences were found in all species in different sampling sites (*p* > 0.05; Table S2).

Statistically significant differences in metal concentrations between seasons were found primarily in the fish’s kidneys or livers, particularly in *C. carpio*. Zn levels in the livers and kidneys were significantly higher in the spring (*C. carpio*) and winter (*H. molitrix* and *O. niloticus*) (*p* < 0.05). Cu concentrations in the kidneys were highest in all three examined fish during the summer, except for *O. niloticus* livers in the spring (*p* < 0.05). Pb levels in all fish were significantly higher in the summer and spring than in other seasons (*p* < 0.05). Cd levels in all tissues of all fish (except the kidneys of *C. carpio* and *O. niloticus*) did not vary significantly with seasons. The Cd content in the kidneys of *C. carpio* and *O. niloticus* was significantly higher in the summer than in the other seasons.

In terms of spatial variability (Table 2), the concentrations of Zn and Cd in *C. carpio* kidneys and livers were found to be significantly higher (*p* < 0.05) in Nam Dinh province (site 4) than in other areas. Similarly, the liver Cu concentrations of *C. carpio* and *H. molitrix* were significantly higher (*p* < 0.05) in Ha Nam (site 2) and Nam Dinh provinces, respectively, than in other sites. There were significantly higher Pb concentrations in the kidneys of *O. niloticus* (*p* < 0.05) and *H. molitrix* and in the livers of *C. carpio* at site 4 than in other sites. All trace metals in the gills of three tested fish showed no spatial variability (*p* > 0.05).

### 3.3. Biomarkers in Fish Tissues

**Figure 3:**
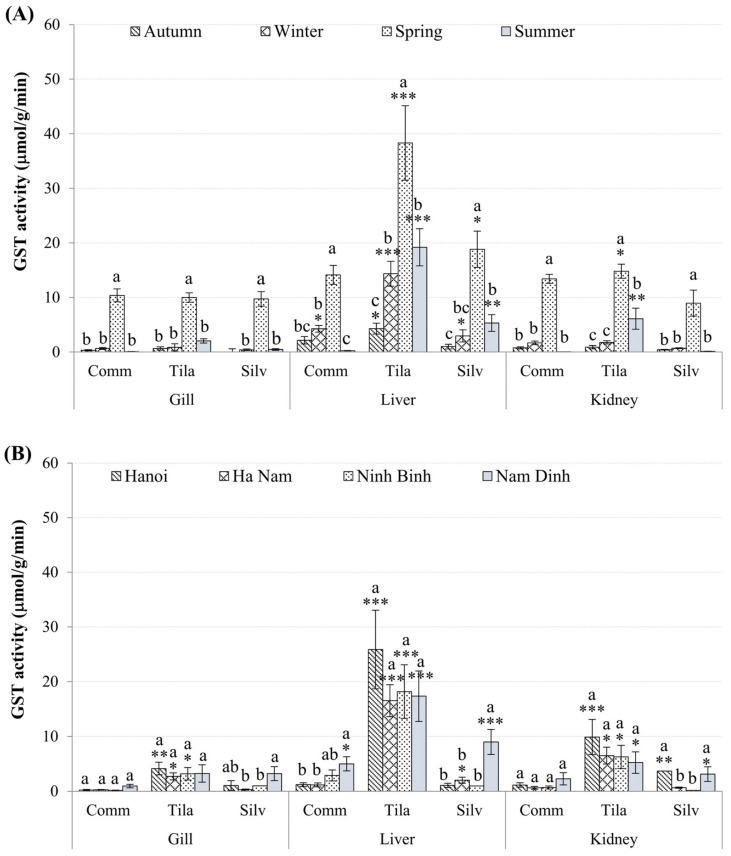
Seasonal (**A**) and spatial (**B**) variation in GST activities (mean ± SEM) in tested tissues of *C. carpio* (Comm), *H. molitrix* (Silv), and *O. niloticus* (Tila) from selected sampling sites. Significant differences in GST levels, of all tissues of three fish species, between sampling times (**A**) and sites (**B**) in comparison to the lowest value (*) are shown (mean ± SEM, *n* = 10–20, * *p* < 0.05, ** *p* < 0.01; *** *p* < 0.001). Different letters (a > b > c) indicate that the variation of GST values between seasons (**A**) or between locations (**B**) within the same organ type is significant (*p* < 0.05; ANOVA followed by Tukey–Kramer multiple comparisons test).

Figure 3 depicts the activities of GST enzymes in the gills, livers, and kidneys of three different fish species. Of the three species, *O. niloticus* had significantly higher GST enzyme activities than the other two (*p* < 0.01). Additionally, GST activities were significantly higher in livers than in other tissues (Figure 3A,B).

In terms of seasonal variation, GST activities were significantly higher in spring (*p* < 0.01) than in other seasons, with GSTs in the livers of *O. niloticus*, *H. molitrix*, and *C. carpio* being 38 ± 6.8, 19 ± 3.3, and 14 ± 1.7 μmol/g wwt./min, respectively. In terms of spatial variability, the highest GST levels were found in Nam Dinh (*p* < 0.0001) for *C. carpio* gills and kidneys, as well as *H. molitrix* livers. When other tissues and species were compared across sites, there was no significant spatial variability in GST activities (*p* > 0.05; Figure 3B).

**Figure 4:**
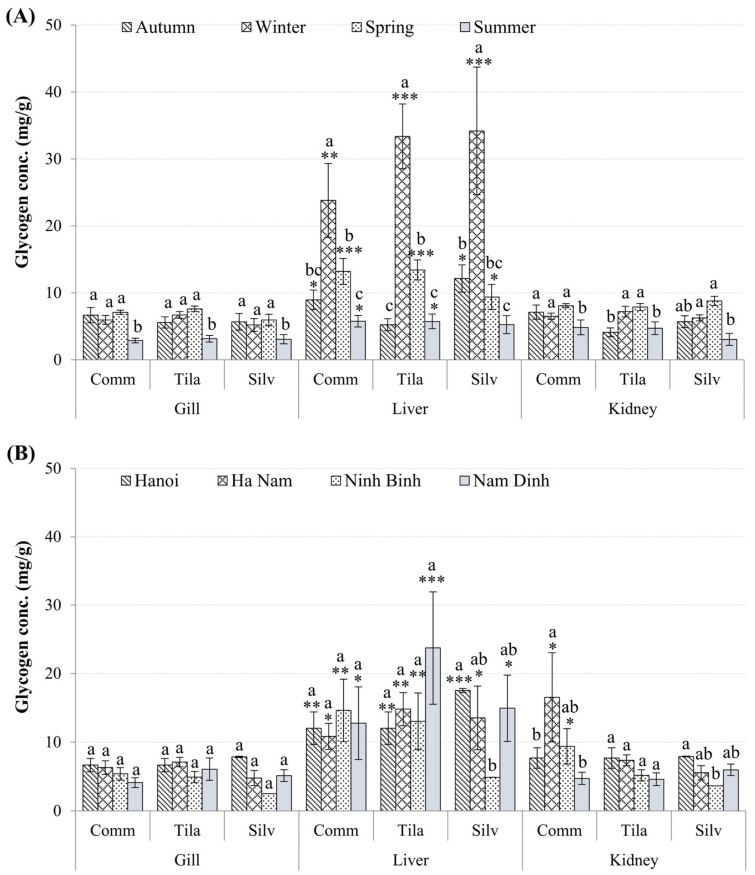
Seasonal and spatial variation in glycogen concentrations (mean ± SEM) in tissues of *C. carpio* (Comm), *H. molitrix* (Silv), and *O. niloticus* (Tila) from selected sampling sites. Significant differences in glycogen levels, of all tissues of three fish species, between sampling times (**A**) and sites (**B**) in comparison to the lowest value (*) are shown (mean ± SEM, *n* = 10–20, * *p* < 0.05, ** *p* < 0.01; *** *p* < 0.001). Different letters (a > b > c) indicate that the variation of glycogen values between seasons (**A**) or between locations (**B**) within the same organ type is significant (*p* < 0.05; ANOVA followed by Tukey–Kramer multiple comparisons test).

Unlike GSTs, the levels of glycogen in all fish livers were significantly higher than in other tissues, especially in the winter (Figure 4A; *p* < 0.01). The glycogen contents of the gills and kidneys did not differ significantly between seasons (*p* > 0.05; Figure 4A), except for higher kidneys glycogen observed in *O. niloticus* in the winter and spring (*p* < 0.05; Figure 4A). Glycogen levels in fish tissue did not differ between species or between study sites (*p* > 0.05), but liver glycogen levels were higher in all three species at all sampling sites (*p* < 0.05; Figure 4B).

**Figure 5:**
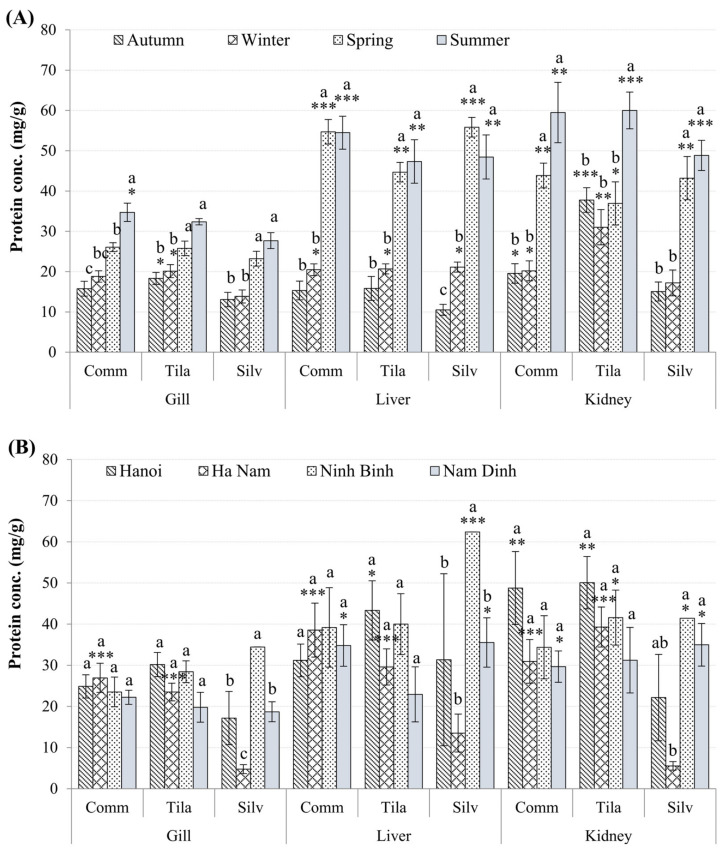
Seasonal and spatial variation in protein concentrations (mean ± SEM) in tissues of the *C. carpio* (Comm), *H. molitrix* (Silv), and *O. niloticus* (Tila) from selected sampling sites. Significant differences in protein levels, of all tissues of three fish species, between sampling times (**A**) and sites (**B**) in comparison to the lowest value (*) are shown (mean ± SEM, n = 10–20, * *p* < 0.05, ** *p* < 0.01; *** *p* < 0.001). Different letters (a > b > c) indicate that the variation of protein values between seasons (**A**) or between locations (**B**) within the same organ type is significant (*p* < 0.05; ANOVA followed by Tukey–Kramer multiple comparisons test).

The protein contents of all tested fish were significantly higher in the livers and kidneys than in the gills (*p* < 0.05; Figure 5A,B), in the summer and spring, compared to other seasons of the year (*p* < 0.05; Figure 5A). There were almost no differences in protein levels between sampling sites (*p* > 0.05), except for lower gill and kidney protein levels in *H.* *molitrix* in Ha Nam compared to other provinces (*p* < 0.01; Figure 5B). However, clear significant differences in protein levels, of all tissues of three fish species were observed between sampling times (Figure 5A) and sites (Figure 5B).

### 3.4. Correlation Test Results

Correlations between metal concentrations in water and those bioaccumulated in fish tissues, as well as between biomarkers (GSTs, glycogen, and protein contents) and metal concentrations (Cu, Zn, Cd, and Pb) in tissues, were examined. Tests for the correlation of metal levels in water and their accumulation in fish tissue revealed no correlation for Cd, only two positive correlations for Zn in kidneys, and the other four for Cu and Pb (Table S1). Among the three tested fish, *O. niloticus* had the most correlations between metals in the water and metals in the fish’s tissues; there were two positive correlations between Cu concentration in the water and its bioaccumulation in the fish’s gills and kidneys in the fall and summer, with *p* = 0.015 and *p* = 0.03, respectively. Furthermore, two linear correlations (*p* < 0.05) were detected between Pb levels in water and its accumulation in the livers (autumn) and gills (winter) of *O. niloticus*. In contrast, only one positive correlation for Cu was found in the livers of *H. molitrix* in the winter (*p* < 0.05). For *C. carpio*, significant correlations were observed primarily for Pb in the summer (gills and livers); in spring, one positive correlation for Zn and one for Cu was found in the kidneys (Table S1).

In *C. carpio*, hepatic Zn levels correlated with its GST activity in both the winter and spring (with correlation coefficient (r) = 0.59, *p* = 0.04 and r = −0.69, *p* = 0.02, respectively), as did hepatic glycogen contents in spring (r = 0.95, *p* = 0.001). More correlations were observed in the kidneys: kidney Cd vs. kidney GST in the autumn (r = 0.7, *p* = 0.03), kidney Pb vs. kidney GST in the winter (r = 0.6, *p* = 0.026), kidneys’ Pb vs. their protein content in the autumn (r = 0.6, *p* = 0.026), and kidneys’ Pb vs. their protein levels in the spring (r = −0.71, *p* = 0.047).

In *O. niloticus*, there were no significant correlations between biomarkers and metal concentrations in the gills (*p* > 0.05). Two correlations observed in the summer, including the liver’s Cu vs. its GSTs (r = −0.53, *p* = 0.04) and the liver’s Cd vs. its protein contents (r = −0.06, *p* = 0.03). Three other correlations observed in spring: the liver’s Pb vs. its protein content (r = 0.54, *p* = 0.049), the kidney Cd vs. kidney protein content (r = −0.7, *p* = 0.01) and the kidney Pb vs. kidney protein content (r = −0.7, *p* = 0.01).

In contrast, correlations were observed in all tissue of *H. molitrix*, such as Zn levels being negatively correlated with GST activities in gills in spring (r = −0.86, *p* = 0.01), Cu and Cd contents being positively correlated with GST activities in livers (r = 0.88, *p* = 0.048, and r = 0.97, *p* = 0.033, respectively). Significant correlations for glycogen include gills’ Cu vs. gills’ glycogen content in the summer (r = −0.91, *p* = 0.032), kidney Cu vs. kidney glycogen content in the winter (r = −0.98, *p* = 0.023), and in the summer (r = 0.97, *p* = 0.033).

## 4. Discussion

### 4.1. Metals in Water and Their Bioaccumulation in Fish

The unexpectedly high levels of almost all metals upstream (site 1, the Red River section; Figure 2A) could be explained by the fact that this river section received effluent from three industrial zones just a few kilometers away, namely Thang Long, Quang Minh, and Noi Bai. Many industries and traditional craft villages (site 1) may contribute to metal pollution in this area. The decrease in metals from site 1 to sites 2 and 3 could be attributed to dilution from less polluted water at the confluence of the Day and Hoang Long rivers, as well as the rivers’ ability to self-clean [31]. The higher Cu and Pb levels observed at site 4 than at other studied sites could be attributed to effluents from several industrial parks, e.g., Dong Van, Kim Binh, Thanh Liem, etc., along the rivers’ basin.

Metal levels varied seasonally in both river and pond water, with the tendency for higher concentrations in the warm season (*p* < 0.05; Figure 2B). Because a large portion of the water in aquaculture ponds came from rivers, the levels of metals in the pond water changed with the levels in the rivers, especially in the case of Cd and Pb. The general low metal concentrations in the winter could be attributed to the low water level of branch rivers from Hanoi during the dry season, i.e., the highly polluted To Lich river did not flow into the Nhue river in the winter [32]. Peaks in metal levels can be caused by industrial activities along rivers as well as changes in water’s physicochemical properties, such as pH, temperature, water hardness, dissolved oxygen, chelating materials, rainfall, and so on [33].

Water quality in the Nhue–Day River basin has deteriorated over the last few decades due to increased urbanization and socioeconomic development. Four metals (Cu, Zn, Cd, and Pb) were discovered to accumulate in the tissues (gills, livers, and kidneys) of three important fish (Table 1 and Table 2). The highest average Zn concentrations were found in all tissues of three fish (85 ± 12 to 303 ± 71 mg/kg wwt. in *C. carpio,* 22 ± 1.75 to 58 ± 6.6 mg/kg wwt. in *H. molitrix,* and 27 ± 2.3 to 91 ± 15 mg/kg wwt. in *O. niloticus*), followed by Cu concentrations. Pb and Cd were accumulated to a much lesser extent in three species. Zn and Cu are essential trace elements that contribute to the synthesis of various vital enzymes and cellular components in living organisms, which explains their high concentrations in fish tissues [34]. Furthermore, because Zn and Cu concentrations in the water were higher than Cd and Pb concentrations (Figure 2), these metals accumulated more readily in fish tissues.

Among all the fish tissues, the livers had significantly higher concentrations of Cu, while the kidneys had higher concentrations of Zn, Pb, and Cd. The livers and kidneys are known as metal target organs because they play an important role in metal detoxification and excretion [35,36], as evidenced by a significantly higher level of detoxification enzyme GSTs observed in the livers, and to a lesser extent in the kidneys (Figure 2). Many proteins in the livers, including hemocuprein and hepatocuprein, as well as numerous oxidative enzymes, require Cu as an essential component for their proper functioning [37]. Furthermore, the presence of other metal-binding proteins, such as metallothionein, which can bind metals for storage and detoxification [38], can result in higher metal concentrations in these organs. Similar findings of metal accumulation patterns in aquatic species have also been reported [36,39]. The current study also discovered inter-species differences in metal accumulation. *C. carpio* showed higher Zn levels in all tissues, whereas *O. niloticus* accumulated more Cu in its livers, which can be explained by a variety of factors, including trophic level, preferred habitat, diet, location, age, and size [40,41]. It has been reported that omnivorous (e.g., *O. niloticus*) or carnivorous fish (e.g., *C. carpio*) tend to accumulate more trace metals than herbivorous ones (e.g., *H. molitrix*) [41]. Similar findings were also reported by Sun and Jeng [42], who reported higher concentrations of Zn in *C. carpio* than in grass carp (*Ctenophryngodon idellus*), *H. molitrix*, or tilapia (*O. mossambicus*). The high levels of metals in *C. carpio* (Zn and Cd) and *O. niloticus* (Cu, Pb, and Cd) might be the reason for more correlations observed in these fish compared to the *H. molitrix* (Table 1, Table 2, and Table S1).

Metal accumulation in fish can also be influenced by seasonal, rather than annual change. Cu and Cd concentrations were significantly higher in the kidneys of *O. niloticus* and *C. carpio*, respectively, in the summer, while Pb concentrations were significantly higher in the livers of *O. niloticus* in spring. This is clearly demonstrated by the responses of various antioxidative and physiological biomarkers, which can be seen through their correlations with trace element accumulation in the tissues in question (Figure 3, Figure 4, Figure 5; Table S1). Several factors, such as differences in growth, reproductive cycles, or changes in water quality parameters, are known to contribute to seasonal variability in metal accumulation in fish [43]. Increased metal levels in fish tissues during the summer or spring (warmer seasons in Vietnam) can be attributed to increased physiological activity of the fish or increased evaporation, hence decreased water level [44]. Köck et al. [45] also discovered higher rates of Cd and Pb uptake in the livers and kidneys of *Salvelinus alpinus* in the summer. Higher metal accumulation has also been reported in various aquatic species in many regions during warmer seasons [46]. Metal concentrations in the kidneys and livers of *C. carpio* and *H. molitrix,* as well as the kidneys of *O. niloticus* were significantly higher in Nam Dinh province than in other areas, indicating spatial variability. This could be due to the high levels of some metals in water, such as Cu and Pb at site 4 (Figure 2), or the fish species inhabiting the area being more susceptible to metal exposure. However, research on the spatial variability of metal accumulation in aquatic animals in Nhue–Day River basins is still scarce. Hence, more research on metal biomonitoring along the two rivers is necessary.

### 4.2. Biomarker Responses in Fish

To counteract the negative effects of ROS and oxidative stress caused by metals in the aquatic environment, living organisms have evolved several antioxidant enzymatic defensive systems [11]. Seasonal variation in the enzyme GST was observed in this study, with hepatic enzymatic activities in all fish tissues significantly higher in the spring than in other seasons (Figure 3), which corresponds to higher levels of trace metal in this tissue (Table 1). This is an early warning sign of fish health deterioration and evidence of chemical stress-related adverse effects. GSTs are a family of enzymes that catalyze the conjugation of reduced glutathione (GSH) with compounds containing reactive electrophilic groups, such as metals or pesticides [12]. In addition to playing crucial roles in the detoxification of xenobiotic compounds and the prevention of oxidative damage, these enzymes are also involved in the repair of oxidized macromolecules and the biosynthesis of physiologically significant metabolites [47,48]. It has been reported that environmental and biological factors such as food availability and reproductive stage can have a significant impact on seasonal variability in metal levels as well as antioxidant defenses [49]. The higher GST activity observed in this study during spring is most likely related to the period of restoration and food availability at this time of year, hence metal bioaccumulation. Current findings showed that during spring, there was a strong positive correlation between hepatic GST activity and hepatic Cu (r = 0.88) or Cd levels (r = 0.97) in the *H. molitrix*. GST induction has also been reported in several studies on various aquatic species, e.g., increased GST activity in all seasons (except spring) in *Leuciscus alburnoides* captured in the Cu mining area of Portugal [50]. According to the author, Cu can cause lipid peroxidation, and the resulting products are GST substrates. A similar response was observed in the livers and kidneys of *O. mossambicus* under sublethal Cd^2+^ exposure conditions (5 mg L^−1^ or 1/10th of LC_50_/48 h) [51]. Nonetheless, a decrease in GST activity may be observed when the organisms are subjected to oxidative stress. This was demonstrated by negative correlations between gill GST activity and gill Zn levels during spring in *H. molitrix* (r = −0.86) and hepatic GST activity vs. hepatic Cu contents during summer in *O. niloticus* (r = −0.53). GST enzymatic activity decreases could be attributed to inactivation by ROS generated by high levels of toxicants [52], which can lead to a reduction in fish’s ability to perform defense reactions. Amado et al. [53] discovered a decrease in GST activity in *Micropogonias furnieri* from the Cu-polluted Patos Lagoon estuary during the winter and summer. Similarly, Oliva et al. [54] also found a negative correlation between GST activity vs. moderately labile inorganic fraction and total Zn concentration in the winter. Moreover, *C. carpio* exposed to 10–100 μM Zn also showed a slight reduction in gill GST activity, indicating a decreased ability to detoxify toxicants [55].

Metal exposure can cause serious biochemical and physiological changes in living organisms, as well as changes in antioxidant defense systems. Under metal stress, fish mobilize their energy reserves to meet the increased demands for detoxification and repair mechanisms, resulting in less energy for normal activities [56]. Because glycogen is the most accessible energy reserve, it is mobilized before lipids or proteins [57]. The current study found seasonal variability in fish glycogen levels, with higher levels in the livers of all tested fish during the winter. Decreases in livers glycogen stores in other seasons could be attributed to the effects of metals; however, no correlation between hepatic livers glycogen and metal levels was found, except for a positive correlation between glycogen and Zn levels in the livers of *C. carpio*. Several previous studies have found that metal exposure can deplete glycogen. For instance, *C. carpio* exposed to a sublethal concentration of Cu (0.08 mg L^−1^ at pH 6.0, 7.5, and 9.0) showed a gradual rise in the whole body’s glucose level over time, which coincided with a decrease in glycogen level, indicating glycogenolysis [58]. It was proposed that energy resources be shifted from reproduction to tolerance mechanisms [59]. High glycogen levels in the winter and low in the summer (Table 1) correspond to low metal levels in the winter and high in the summer (Figure 3), suggesting that fish in the Nhue–Day River basin have very high energy demand (mobilized from glycogen reserves) to combat with metal stress, in addition to body condition factors.

Aside from glycogen reserves, total protein content in tissues is widely regarded as an animal health indicator [60]. When exposed to trace metals, changes in protein metabolism, such as increased protein synthesis or breakdown and inhibition or activation of specific enzymes, may occur in living organisms [61]. In the current study, total livers and kidney proteins were found to be higher in all fish species during the spring and summer. In addition, both *C. carpio* and *O. niloticus* had negative correlations between kidney protein levels and kidney Pb levels during the spring. When exposed to metals, protein catabolism can occur, in which free amino acids are mobilized to meet extra energy demands for the organisms [62]. Several studies have found that metal exposure reduces protein levels in muscles and liver tissues [56,62]. Current findings suggest that Pb may exert a negative impact on protein metabolism in fish. However, positive correlations between kidney Pb levels and kidney protein contents were found in autumn in *C. carpio* (r = 0.61) and spring in *O. niloticus* (r = 0.66). This could be that fish in the Nhue–Day River induced MT synthesis to counteract stresses caused by Cd or Pb, allowing more metals to be bound and stored in target tissues for cellular protection against toxicity [63].

In summary, metal pollution alters the antioxidant enzymes (GSTs) in various fish tissues, particularly the livers and kidneys of all tested fish. Changes in GSTs induced the mobilization of energy reserves (protein and glycogen) toward tolerance of oxidative stressors, i.e., metals, resulting in a decrease in energy available for normal functions of organisms when stress levels increased. The higher GST levels in this study suggest roles as protective biomarkers (e.g., xenobiotic biotransformation and ROS scavenging) and reveal the response of these fish to chemical stress [64]. However, once the metal concentrations reach a certain level, enzyme activity is reduced due to protein damage at higher metal concentrations. The current findings also show a clear link between metal pollution in the aquatic environment and antioxidant biomarkers, followed by energy reserves.

## 5. Conclusions

Metal concentrations in the water of the rivers and aquaculture ponds varied over time and space (*p* < 0.05), were higher at sites 1 and 4 (than at other sites), and increased during the warm seasons. These metals accumulated more in the livers and kidneys of tested fish, particularly *C. carpio* (Zn and Cd) and *O. niloticus* (Cu, Pb, and Cd). This tendency was seen again in the correlations between metal levels in water and fish tissues, which was prominent in tilapia, then common carp.

Similarly, biochemical and physiological biomarkers were altered over time and in different ways. Specifically, higher GST levels were observed in the spring, higher glycogen levels in the winter, and higher protein levels in the spring and summer. Fish in the river basin demonstrated high energy demand, an early indicator of fish health deterioration and evidence of chemical stress-related adverse effects. Some correlations were found between metal concentrations in water and their accumulation in fish tissues, as well as between biomarkers (GSTs, glycogen, protein) and tissue metal concentrations. This implies that metal levels in the water have reached levels that impact the biochemical and physiological biomarkers of fish in the river basin and, hence, fish health.

Despite the complexity of the biomarker response, the current findings demonstrate a clear linkage between metal pollution in the aquatic environment and the response of biochemical and physiological biomarkers in fish living in that environment. This biomarker set can be used as an effective indicator for the early warning of stress in fish caused by metal toxicity and assess the health status of exposed organisms. This set of biomarkers has been tested and evaluated in different seasons (weather) and over a wide geographical area; therefore, it can also be used as a tool for environmental monitoring and river basin management.

## Figures and Tables

**Table 1:** Metal concentrations in the gills, livers, and kidneys of *C. carpio*, *H. molitrix*, and *O. niloticus* collected over four seasons. The data are presented as mean ± SEM. Small letters (a > b) represent statistically significant differences between seasons within the same tissue samples from the same species. The capital letters (A > B > C) represent statistically significant differences between tissues of each species within each season.

	*C. carpio*			*H. molitrix*			*O. niloticus*		
	Gills	Livers	Kidneys	Gills	Livers	Kidneys	Gills	Livers	Kidneys
Zn concentration (mg kg^−1^ wet weight)									
Spring	227 ± 29 ^aA^	189 ± 60 ^bA^	303 ± 71 ^bA^	30 ± 6.5 ^aA^	44 ± 6.7 ^aA^	37 ± 8.2 ^abA^	29 ± 3.4 ^abA^	31 ± 2.9 ^aA^	47 ± 4.1 ^aA^
Summer	183 ± 23 ^aB^	85 ± 12 ^aA^	230 ± 42 ^abB^	30 ± 3.2 ^aA^	41 ± 7.6 ^aA^	31 ± 1.5 ^aA^	28 ± 1.6 ^aA^	38 ± 3.7 ^aA^	63 ± 12 ^abB^
Autumn	133 ± 28 ^aA^	88 ± 15 ^aA^	140 ± 18 ^aA^	22 ± 1.7 ^aA^	46 ± 8.3 ^aB^	29 ± 5.0 ^aAB^	27 ± 2.3 ^aA^	32 ± 2.8 ^aA^	46 ± 9.0 ^aAB^
Winter	200 ± 21 ^aAB^	109 ± 14 ^abA^	236 ± 38 ^abB^	32 ± 8.8 ^aA^	58 ± 6.6 ^aB^	49 ± 10.4 ^bB^	38 ± 4.9 ^abA^	36 ± 3.0 ^aA^	91 ± 15 ^bB^
**Average**	**190 ± 25**	**118 ± 25**	**227 ± 42**	**28 ± 5.1**	**47 ± 7.3**	**37 ± 6.3**	**30 ± 3.1**	**34 ± 3.1**	**62 ± 10**
Cu concentration (mg kg^−1^ wet weight)									
Spring	1.7 ± 0.21 ^aA^	16.1 ± 7.1 ^abB^	6.2 ± 1.5 ^abB^	2.0 ± 0.37 ^abA^	34 ± 14 ^aB^	2.9 ± 0.43 ^aA^	2.3 ± 0.14 ^aA^	204 ± 37 ^bC^	5.2 ± 0.48 ^aB^
Summer	3.5 ± 0.21 ^bA^	16.8 ± 1.9 ^abB^	10.7 ± 1.3 ^bAB^	4.5 ± 0.89 ^bA^	31 ± 6.7 ^aC^	8.8 ± 0.69 ^bB^	3.6 ± 0.16 ^bA^	100 ± 13 ^aC^	13 ± 2.2 ^bB^
Autumn	1.5 ± 0.15 ^aA^	12.0 ± 2.5 ^aB^	4.0 ± 0.41 ^aA^	0.93 ± 0.08 ^aA^	23 ± 7.9 ^aB^	1.7 ± 0.27 ^aA^	1.3 ± 0.10 ^aA^	80 ± 18 ^aC^	3.5 ± 0.52 ^aB^
Winter	1.2 ± 0.11 ^aA^	24 ± 5.6 ^bC^	4.4 ± 0.36 ^aB^	1.3 ± 0.19 ^aA^	21 ± 6.8 ^aC^	3.6 ± 0.68 ^abB^	1.7 ± 0.19 ^aA^	101 ± 10 ^aC^	5.7 ± 0.63 ^aB^
**Average**	**1.9 ± 0.17**	**17 ± 4.2**	**6.3 ± 0.91**	**2.2 ± 0.38**	**27 ± 8.8**	**4.3 ± 0.52**	**2.2 ± 0.15**	**121 ± 20**	**6.9 ± 1.0**
Pb concentration (mg kg^−1^ wet weight)									
Spring	0.73 ± 0.11 ^aA^	0.39 ± 0.05 ^abA^	1.6 ± 0.36 ^bB^	1.1 ± 0.34 ^bA^	0.76 ± 0.12 ^abA^	1.2 ± 0.56 ^bA^	0.77 ± 0.09 ^bA^	0.93 ± 0.13 ^abA^	1.1 ± 0.16 ^abA^
Summer	0.57 ± 0.04 ^aA^	0.75 ± 0.06 ^bA^	1.2 ± 0.17 ^bB^	0.74 ± 0.16 ^abA^	1.58 ± 0.57 ^bA^	1.6 ± 0.63 ^bA^	0.62 ± 0.04 ^bA^	1.4 ± 0.22 ^bB^	1.3 ± 0.21 ^bB^
Autumn	0.50 ± 0.08 ^aA^	0.23 ± 0.05 ^aA^	0.29 ± 0.06 ^aA^	0.31 ± 0.05 ^aA^	0.29 ± 0.07 ^aA^	0.25 ± 0.06 ^aA^	0.61 ± 0.08 ^abA^	0.45 ± 0.08 ^aA^	1.2 ± 0.29 ^bB^
Winter	0.48 ± 0.10 ^aA^	0.24 ± 0.04 ^aA^	0.33 ± 0.05 ^aA^	0.28 ± 0.04 ^aA^	0.28 ± 0.04 ^aA^	0.27 ± 0.08 ^aA^	0.31 ± 0.04 ^aA^	0.63 ± 0.14 ^aA^	0.59 ± 0.11 ^aA^
**Average**	**0.57 ± 0.084**	**0.40 ± 0.049**	**0.86 ± 0.16**	**0.61 ± 0.15**	**0.73 ± 0.20**	**0.85 ± 0.33**	**0.58 ± 0.063**	**0.86 ± 0.14**	**1.0 ± 0.19**
Cd concentration (μg kg^−1^ wet weight)									
Spring	6.3 ± 2.6 ^aA^	98 ± 42 ^aA^	220 ± 68 ^aA^	4.0 ± 2.4 ^aA^	46 ± 32 ^aA^	116 ± 54 ^aA^	4.3 ± 1.7 ^aA^	211 ± 40 ^aA^	373 ± 72 ^aA^
Summer	89 ± 9.3 ^aA^	105 ± 10 ^aA^	435 ± 65 ^aA^	60 ± 10 ^aA^	97 ± 36 ^aA^	162 ± 25 ^aA^	71 ± 6.8 ^aA^	269 ± 44 ^aA^	311 ± 43 ^aA^
Autumn	5.9 ± 1.0 ^aA^	16 ± 2.3 ^aA^	99 ± 13 ^aA^	5.9 ± 2.0 ^aA^	23 ± 6.0 ^aA^	113 ± 33 ^aA^	8.8 ± 1.8 ^aA^	82 ± 18 ^aA^	219 ± 49 ^aA^
Winter	3.1 ± 1.0 ^aA^	30 ± 4.2 ^aA^	158 ± 25 ^aA^	6.2 ± 3.6 ^aA^	52 ± 29 ^aA^	305 ± 86 ^aA^	2.9 ± 0.5 ^aA^	165 ± 33 ^aA^	234 ± 57 ^aA^
**Average**	**25 ± 3.5**	**62 ± 15**	**288 ± 43**	**19 ± 4.6**	**54 ± 2.6**	**174 ± 49**	**22 ± 2.7**	**182 ± 34**	**284 ± 55**

**Table 2:** Metal concentrations in the gills, livers, and kidneys of *C. carpio*, *H. molitrix*, and *O. niloticus* collected from different sites. The data are presented as mean ± SEM. Small letters (a > b > c) represent statistically significant differences between sampling sites within the same tissue samples from the same species. The capital letters (A > B > C) represent statistically significant differences between tissues of each species within each site.

	*C. carpio*			*H. molitrix*			*O. niloticus*		
	Gills	Livers	Kidneys	Gills	Livers	Kidneys	Gills	Livers	Kidneys
Zn concentration (mg kg^−1^ wet weight)									
Site 1	222 ± 19 ^aA^	114 ± 14 ^abB^	168 ± 19 ^bAB^	21 ± 5.1 ^bA^	31 ± 4.4 ^bA^	22 ± 1.8 ^bA^	37 ± 4.6 ^aB^	37 ± 3.2 ^aB^	72 ± 12 ^aA^
Site 2	145 ± 26 ^bAB^	82 ± 15 ^abB^	206 ± 45 ^abA^	22 ± 2.8 ^bB^	54 ± 7.5 ^aA^	40 ± 6.1 ^aAB^	32 ± 2.9 ^abB^	42 ± 4.9 ^aAB^	62 ± 13 ^abA^
Site 3	156 ± 29 ^aA^	59 ± 9.5 ^bB^	142 ± 30 ^bA^	31 ± 3.8 ^aA^	34 ± 5.5 ^abA^	35 ± 6.4 ^aA^	26 ± 1.3 ^bC^	34 ± 2.1 ^abB^	48 ± 4.6 ^bA^
Site 4	196 ± 21 ^aAB^	132 ± 22 ^aB^	313 ± 40 ^aA^	28 ± 2.8 ^abB^	45 ± 5.1 ^aA^	36 ± 5.8 ^aAB^	25 ± 2.4 ^bB^	28 ± 1.4 ^bB^	77 ± 17 ^aA^
**Average**	**184 ± 13**	**109 ± 11.5**	**243 ± 25**	**27 ± 2.3**	**56 ± 10**	**50 ± 13.8**	**35 ± 3.5**	**42 ± 4.1**	**83 ± 10.8**
Cu concentration (mg kg^−1^ wet weight)									
Site 1	2.2 ± 0.27 ^aC^	18 ± 2.5 ^abA^	6.5 ± 0.94 ^aB^	1.2 ± 0.28 ^cB^	7.4 ± 2.8 ^bA^	1.8 ± 0.12 ^cB^	2.5 ± 0.30 ^aC^	124 ± 18 ^aA^	6.4 ± 1.2 ^bB^
Site 2	2.6 ± 0.32 ^aC^	22 ± 4.7 ^aA^	6.6 ± 1.1 ^aB^	1.3 ± 0.23 ^cC^	22 ± 2.7 ^abA^	3.6 ± 0.89 ^bB^	2.7 ± 0.48 ^aC^	113 ± 13 ^aA^	8.3 ± 2.1 ^abB^
Site 3	2.2 ± 0.46 ^aC^	12 ± 3.7 ^abA^	6.1 ± 1.2 ^aB^	3.8 ± 3.2 ^aB^	35 ± 7.1 ^aA^	9.5 ± 3.1 ^aB^	2.3 ± 0.35 ^aC^	98 ± 25 ^aA^	6.8 ± 1.1 ^abB^
Site 4	2.2 ± 0.41 ^aC^	12 ± 2.3 ^bA^	4.9 ± 0.78 ^aB^	2.0 ± 0.38 ^bB^	27 ± 6.5 ^aA^	4.4 ± 0.94 ^abB^	2.7 ± 0.44 ^aC^	106 ± 26 ^aA^	11 ± 2.8 ^aB^
**Average**	**2.3 ± 0.20**	**20 ± 3.1**	**6.4 ± 0.60**	**1.8 ± 0.24**	**20 ± 3.1**	**4.1 ± 0.63**	**2.6 ± 0.22**	**107 ± 15.5**	**8.9 ± 1.8**
Pb concentration (mg kg^−1^ wet weight)									
Site 1	0.54 ± 0.07 ^aA^	0.41 ± 0.07 ^abA^	0.67 ± 0.16 ^abA^	0.29 ± 0.20 ^bA^	0.10 ± 0.05 ^bA^	0.12 ± 0.03 ^cA^	0.69 ± 0.09 ^aA^	0.97 ± 0.13 ^aA^	0.87 ± 0.09 ^abA^
Site 2	0.52 ± 0.07 ^aA^	0.54 ± 0.08 ^aA^	0.64 ± 0.14 ^bA^	0.31 ± 0.04 ^bA^	0.32 ± 0.07 ^abA^	0.32 ± 0.07 ^bA^	0.58 ± 0.07 ^aB^	0.76 ± 0.12 ^abAB^	1.3 ± 0.25 ^abA^
Site 3	0.45 ± 0.12 ^aA^	0.50 ± 0.14 ^aA^	0.93 ± 0.23 ^aA^	0.5 ± 0.01 ^abB^	0.68 ± 0.15 ^aAB^	1.0 ± 0.13 ^aA^	0.50 ± 0.08 ^aA^	0.58 ± 0.12 ^bA^	0.80 ± 0.16 ^bA^
Site 4	0.63 ± 0.07 ^aA^	0.45 ± 0.09 ^aA^	0.83 ± 0.18 ^aA^	0.75 ± 0.16 ^aA^	0.73 ± 0.12 ^aA^	0.72 ± 0.17 ^abA^	0.67 ± 0.20 ^aB^	0.74 ± 0.17 ^abAB^	1.6 ± 0.42 ^aA^
**Average**	**0.57 ± 0.05**	**0.47 ± 0.045**	**0.90 ± 0.12**	**0.56 ± 0.17**	**0.53 ± 0.08**	**0.53 ± 0.33**	**0.63 ± 0.09**	**0.88 ± 0.10**	**1.3 ± 0.24**
Cd concentration (μg kg^−1^ wet weight)									
Site 1	28 ± 8.9 ^aB^	48 ± 12 ^bB^	203 ±51 ^bA^	4.5 ± 2.5 ^cC^	12 ± 1.0 ^bB^	125 ±6.0 ^bA^	37 ± 14 ^aC^	195 ± 32 ^abB^	402 ±85 ^aA^
Site 2	34 ± 10 ^aC^	70 ± 17 ^abB^	189 ± 37 ^bA^	21 ± 8.0 ^bB^	47 ± 13 ^aB^	186 ± 47 ^aA^	30 ± 9.0 ^aB^	255 ± 40 ^aA^	320 ± 50 ^abA^
Site 3	8.9 ± 6.0 ^bC^	31 ± 8.2 ^bB^	196 ± 55 ^bA^	50 ± 9.8 ^aB^	54 ± 7.5 ^aB^	200 ± 61 ^aA^	17 ± 6.0 ^bC^	123 ± 27 ^bB^	204 ± 37 ^bA^
Site 4	12 ± 4.5 ^bC^	97 ± 23 ^aB^	461 ± 115 ^aA^	19 ± 6.3 ^bC^	58 ± 17 ^aB^	148 ± 42 ^abA^	29 ± 13 ^abC^	124 ± 31 ^bB^	259 ± 65 ^bA^
**Average**	**25 ± 5.3**	**70 ± 10**	**259 ± 83**	**24 ± 6.1**	**49 ± 9.8**	**162 ± 27**	**23 ± 4.6**	**190 ± 22**	**308 ± 33**

Notes: Site 1: Hanoi; Site 2: Ha Nam; Site 3: Ninh Binh; Site 4: Nam Dinh.

## Data Availability

Data sharing is not applicable to this article.

## Supplementary Materials

The following supporting information can be downloaded at: https://www.mdpi.com/article/10.3390/toxics10090528/s1, Table S1. Observed significant correlations (*p* < 0.05) between metal concentrations in water and their accumulation in tissues of common carp (*Cyprinus carpio*), silver carp (*Hypothalmic molitrix*), and tilapia (*Oreochromis niloticus*) for study seasons and regions; Table S2. Annual metals variation in the gills, livers, and kidneys of common carp (*Cyprinus carpio*), silver carp (*Hypothalmic molitrix*), and tilapia (*Oreochromis niloticus*) collected from different sites. The data is presented as mean ± SEM. Small letters (a > b) represent statistically significant differences between sampling years within the same tissue samples from the same species. The capital letters (A > B > C) represent statistically significant differences between tissues of each species within each year.
