# Supplementary material for: Adverse Effects of Toxic Metal Pollution in Rivers on the Physiological Health of Fish
Source: Toxics. 2022 Sep 8;10(9):528. doi: 10.3390/toxics10090528 (PMC9502420; doi:10.3390/toxics10090528)
Supplement: Supplementary file 1 [file toxics-10-00528-s001.zip › toxics-1866577-supplementary.pdf]

Table S1. Observed significant correlations ($p < 0.05$) between metal concentrations in water and their accumulation in tissues of common carp (*Cyprinus carpio*), silver carp (*Hypothalmic molitrix*), and tilapia (*Oreochromis niloticus*) for study seasons and regions

		**Zn**			**Cu**			**Cd**			**Pb**		
		G	L	K	G	L	K	G	L	K	G	L	K
Common carp	Autumn	-	-	-	-	-	-	-	-	-	-	-	-
	Winter	-	-	-	-	-	-	-		-	-	-	-
	Spring	-	-	+	-	-	+	-	-	-	-	-	-
	Summer	-	-	-	-	-	-	-	-	-	+	+	-
Silver carp	Autumn	-	-	-	-	-	-	-	-	-	-	-	-
	Winter	-	-	-	-	+	-	-	-	-	-	-	-
	Spring	-	-	-	-	-	-	-	-	-	-	-	-
	Summer	-	-	-	-	-	-	-	-	-	-	-	-
Tilapia	Autumn	-	-	+	+	-	-	-	-	-	-	+	-
	Winter	-	-	-	-	-	-	-	-	-	+	-	-
	Spring	-	-	-	-	-	-	-	-	-	-	-	-
	Summer	-	-	-	-	-	+	-	-	-	-	-	-
Common carp	Ha Noi	-	-	-	-	-	-	+	-	-	-	-	+
	Ha Nam	-	-	-	-	-	-	-	-	+	-	-	+
	Ninh Binh	-	-	-	-	-	-	+	+	-	-	-	-
	Nam Dinh	-	-	-	-	-	+	+	-	-	+	-	-
Silver carp	Ha Noi	-	-	-	-	-	-	-	-	-	-	-	-
	Ha Nam	-	-	-	-	-	-	+	-	-	-	+	-
	Ninh Binh	-	-	-	-	-	-	-	-	-	-	-	-
	Nam Dinh	-	-	-	-	-	-	-	-	-	-	-	-
Tilapia	Ha Noi	-	-	-	-	-	-	-	-	-	-	-	-
	Ha Nam	-	-	+	-	-	-	-	-	-	-	+	+
	Ninh Binh	-	-	-	-	-	-	+	-	-	-	-	-
	Nam Dinh	-	-	-	-	-	-	+	-	-	-	-	-

Notes: G: gills; L: livers; K: kidneys; Correlation (+); no correlation (-)

Table S2. Annual metals variation in the gills, livers, and kidneys of common carp (*Cyprinus carpio*), silver carp (*Hypothalmic molitrix*), and tilapia (*Oreochromis niloticus*) collected from different sites. The data is presented as mean ± SEM. Small letters (a > b) represent statistically significant differences between sampling years within the same tissue samples from the same species. The capital letters (A > B > C) represent statistically significant differences between tissues of each species within each year.

	C. carpio		***H. molitrix***		***O. niloticus***	
	The year 2013	The year 2014	The year 2013	The year 2014	The year 2013	The year 2014
Zn concentration (mg kg^{-1} wet weight)						
Gills	190±25^{aAB}	180±25^{aA}	28±5.1^{aB}	24±4.2^{aB}	30±3.1^{aB}	29±3.0^{aB}
Liver	118±25^{abB}	97±15^{aB}	47±7.3^{aA}	42±5.9^{aA}	35±3.1^{aB}	35±6.1^{aB}
Kidneys	227±42^{aA}	207±34^{aA}	37±6.2^{aAB}	38±9.1^{aAB}	62±10^{aA}	65±12^{aA}
Cu concentration (mg kg^{-1} wet weight)						
Gills	1.9±0.17^{aC}	2.2±0.4^{aC}	2.2±0.38^{aC}	1.5±0.3^{aC}	2.2±0.15^{aC}	2.4±0.3^{aC}
Liver	17±4.1^{aA}	16±3.0^{aA}	27±8.7^{aA}	19±4.0^{aA}	121±10^{aA}	106±20^{aA}
Kidneys	6.2±0.9^{aB}	6.0±1.0^{aB}	4.3±0.51^{aB}	3.5±0.61^{aB}	6.9±1.0^{aB}	10±2.5^{aB}
Pb concentration (mg kg^{-1} wet weight)						
Gills	0.57±0.084^{aAB}	0.52±0.08^{aAB}	0.61±0.15^{aA}	0.52±0.13^{aA}	0.58±0.061^{aB}	0.57±0.07^{aB}
Liver	0.40±0.049^{aB}	0.46±0.10^{aB}	0.73±0.15^{bA}	0.38±0.08^{aA}	0.86±0.14^{aAB}	0.76±0.13^{aAB}
Kidneys	0.86±0.16^{aA}	0.77±0.18^{aA}	0.85±0.31^{aA}	0.68±0.13^{aA}	1.0±0.18^{aA}	1.13±0.22^{aA}
Cd concentration (μg kg^{-1} wet weight)						
Gills	25±3.5^{aC}	21±7.0^{aC}	19±4.0^{aC}	15 ±6.0^{aC}	22±2.4^{aC}	28±11^{aC}
Liver	62±15^{aB}	61±12^{aB}	54±23^{aB}	39±12^{aB}	182±34^{aB}	174±33^{aB}
Kidneys	228±43^{aA}	242±64^{aA}	174±49^{aA}	153±32^{aA}	284±25^{aA}	296±59^{bA}
